# Profiling diverse sequence tandem repeats in colorectal cancer reveals co-occurrence of microsatellite and chromosomal instability involving Chromosome 8

**DOI:** 10.1186/s13073-021-00958-z

**Published:** 2021-09-06

**Authors:** GiWon Shin, Stephanie U. Greer, Erik Hopmans, Susan M. Grimes, HoJoon Lee, Lan Zhao, Laura Miotke, Carlos Suarez, Alison F. Almeda, Sigurdis Haraldsdottir, Hanlee P. Ji

**Affiliations:** 1 Division of Oncology, Department of Medicine, Stanford University School of Medicine, 269 Campus Drive, Stanford, CA 94305-5151 USA; 2 Department of Pathology, Stanford University School of Medicine, Palo Alto, CA 94304 USA; 3 Stanford Genome Technology Center, Stanford University, Palo Alto, CA 94304 USA

**Keywords:** Microsatellite instability, Sequence tandem repeats, Chromosomal instability, Colorectal cancer, DNA mismatch repair

## Abstract

We developed a sensitive sequencing approach that simultaneously profiles microsatellite instability, chromosomal instability, and subclonal structure in cancer. We assessed diverse repeat motifs across 225 microsatellites on colorectal carcinomas. Our study identified elevated alterations at both selected tetranucleotide and conventional mononucleotide repeats. Many colorectal carcinomas had a mix of genomic instability states that are normally considered exclusive. An *MSH3* mutation may have contributed to the mixed states. Increased copy number of chromosome arm 8q was most prevalent among tumors with microsatellite instability, including a case of translocation involving 8q. Subclonal analysis identified co-occurring driver mutations previously known to be exclusive.

**Supplementary Information:**

The online version contains supplementary material available at 10.1186/s13073-021-00958-z.

## Background

Microsatellites are composed of short tandem repeats (STRs) and are present throughout the human genome. STRs have different classes of motifs that include mono-, di-, tri-, and tetranucleotide sequences. In colorectal carcinoma (CRC), somatic mutations or methylation of DNA mismatch repair (MMR) genes (i.e., *MSH2*, *MLH1*, *PMS2*, *MSH6*) lead to increased mutation rates, particularly in microsatellites. Lynch syndrome is an autosomal dominant genetic disorder in which affected individuals are carriers of deleterious germline mutations in the MMR genes and have a substantially increased risk of CRC as well as other malignancies. Somatic inactivation of the remaining wildtype allele of an MMR gene leads to inactivation of this DNA repair pathway and increased risk of developing tumors. Tumors with MMR loss display hypermutability in microsatellite sequences. This phenomenon is referred to as microsatellite instability (MSI) and is characterized by the accrual of insertions or deletions **(**indels) in either coding or non-coding microsatellite sequences. Based on specific criteria, tumors with high levels of microsatellite mutations are referred to as MSI-high (MSI-H) with mutation rates that are orders of magnitude greater than what is observed in tumors that are microsatellite stable (MSS) [1]. Importantly, MSI occurs in all classes of microsatellite repeats. However, nearly all published studies have exclusively focused on the presence of microsatellite mutations within mono- and dinucleotide repeats to assess MSI. Generally, there has not been a careful examination of other microsatellite classes, potentially missing important features of MSI and the underlying genomic complexity of these tumors.

Determining the MSI status of CRCs and other tumors is of increasing importance given advances in cancer immunotherapy. MSI-positive CRCs respond to immune checkpoint therapy while CRCs with CIN do not. MSI-related indels in exons produce frameshift mutations within a gene, leading to a higher number of novel peptides, also referred to as neopeptides, from the translated mutated protein. For MSI-H cancers, these neopeptides provide an abundance of unique cancer antigens that are absent from normal colon and rectal cells. Pembrolizumab and other immune checkpoint drugs stimulate the immune system such that T cells recognize these highly immunogenic cancer cells and their cancer-related neoantigens [2]. Given the predictive nature of MSI status for immunotherapy, molecular genetic testing is a diagnostic requirement for receiving immune checkpoint therapies.

There are a number of methods used for detecting MSI in cancer. One approach involves immunohistochemistry (IHC) staining of tumor sections for MMR protein expression of MLH1, MSH2, MSH6, and PMS2. A tumor lacking expression of one of these proteins is considered to have MSI. The most common molecular genetic assay for identifying MSI-H tumors requires PCR amplification of a limited panel of microsatellite markers. The MSI PCR test uses a multiplexed amplicon assay which requires testing five or more microsatellite markers—typically these are either mono- or dinucleotide repeats [3, 4]. Using capillary electrophoresis (CE), tumor-specific changes in the microsatellite amplicon size indicate MSI when compared to the microsatellite genotypes from matched non-tumor cells. If a sufficient number of microsatellites demonstrate an allelic shift in size (e.g., two or more), the tumor is classified as MSI-H. PCR testing is considered to be the gold standard test for MSI-H. In comparison to MSI PCR, MMR IHC misses approximately 10% of tumors with MSI [5, 6]. Despite its important diagnostic role, MSI PCR tests have issues which include a significant level of artifact mutations from amplification errors.

Next-generation sequencing (NGS) approaches for detecting MSI are based on targeted assays that enrich or amplify exon sequences (e.g., exomes and gene panels) or microsatellites [7–11]. When using targeted sequencing with gene panels and exomes, the presence of indels within exon-based mono- or dinucleotide repeats determines MSI status. Indels in microsatellite tracts lead to allelic shifts and somatic genotypes. Generally, MSI NGS assays have high concordance with MSI PCR tests [7, 9–11]. However, MSI NGS tests are also susceptible to artifacts related to sequencing library amplification. PCR amplification stutter occurs in all types of sequencing data. Therefore, detection of MSI at low tumor cellular fraction remains a challenge for NGS detection.

The traditional criteria for defining MSI are restricted to mono- or dinucleotide repeats. However, there is another MSI category that involves elevated microsatellite alterations at selected tetranucleotide repeats (EMAST). This category of microsatellite alterations is thought to be related to changes in the function of *MSH3*, another gene in the MMR pathway, whereby loss of MSH3 function leads to instability in dinucleotide or longer repeats [12]. CRCs with EMAST have been reported in up to 50% of tumors [12]. The EMAST phenotype may be associated with an elevated microsatellite mutation rate of mono- and dinucleotide repeats, but this is not consistently observed [13–16]. EMAST CRCs are also frequently associated with chromosomal instability (CIN) where portions of the genome show copy number alterations, aneuploidy, and rearrangements [12]. However, there are few, if any, genomic studies that have examined these EMAST CRCs in detail.

Detection of EMAST involves testing a set of tetranucleotide microsatellites for instability changes. The most common method involves PCR genotyping assays analyzed with CE [13]. Currently, there are no established markers or criteria which are used in classifying EMAST [17]. Recent studies have relied on using five or more tetranucleotide markers; a tumor is considered to be EMAST positive when 30% or more of the markers are unstable compared to the matched normal DNA genotypes [13–16]. NGS methods for detecting EMAST are generally not available, since most targeted assays do not include microsatellites, such as tetranucleotide repeats [7–10]. Generally, exons lack tetranucleotide repeats of sufficient length and as a result, exome or targeted gene sequencing will miss these genomic instability features. Furthermore, targeted sequencing assays with short reads may not span the entire length of tetranucleotide microsatellites which are frequently longer than mono- and dinucleotide repeats.

As noted, nearly all studies determining the presence of MSI in CRCs and other cancers examine only mono- and dinucleotide repeats [18]. Limiting the evaluation of MSI to only two classes of microsatellites overlooks more complex genetic features, such as instability in tetranucleotide repeats. Addressing this gap, we developed a new sequencing approach to profile instability across different classes of microsatellites and cancer genes. Our analysis included an expanded set of mono-, di-, tri-, and tetranucleotide repeats with minimal amplification error and high read coverage. We used ultra-high depth sequencing coverage in the thousands for sensitive and specific detection of somatic events such as microsatellite and gene mutations. Thus, one can quantify genomic changes that are indicative of genetic heterogeneity and subclonal diversity present in a given tumor. Simultaneously, we detected CIN via the high-accuracy identification of copy number alterations in cancer genes. As we demonstrate on a cohort of CRCs, this approach provided quantitative microsatellite profiles informative for MSI and EMAST, revealed mixed classes of CIN, and delineated genetic heterogeneity indicative of subclonal structure.

## Methods

### Cancer samples

This study was conducted in compliance with the Helsinki Declaration. All patients were enrolled according to a study protocol approved by the Stanford University School of Medicine Institutional Review Board (IRB-11886 and IRB-48492). Informed consent was obtained from all patients. Tissues were obtained from the Stanford Cancer Institute Tissue bank and the Landspitali University Hospital. For this pilot study, 46 pairs of matched tumor and normal specimens were used for PCR-based MSI tests and various types of sequencing analyses (Additional file 2: Table S1). Ascertainment of samples was based on the availability and cellularity of matched normal tumor samples between 2006 and 2011.

All the specimens underwent histopathology review to mark areas of tumor and normal tissue on hematoxylin and eosin-stained tissue sections and on the corresponding paraffin blocks. The samples were generally 60% tumor purity or higher. We macro-dissected the samples to provide improved tumor purity and extracted genomic DNA from the matched normal and tumor CRC samples. The dissected tissue was homogenized and processed using the E.Z.N.A. SQ RNA/DNA/Protein Extraction Kit (Omega Biotek Inc.). Briefly, we lysed the cells using the provided lysis buffer (SQ1), precipitated and removed proteins with protease (SQ2) and NaOAc, precipitated nucleic acids with isopropanol, washed, and re-suspended nucleic acid pellets in 10 mM Tris-HCl (pH 8.0) buffer. We removed RNA species in the nucleic acid via the addition of 4 μg of RNase A (Promega) and incubation at 37°C for 1 h. After incubation, each sample was purified with AMPure XP beads in a bead solution-to-sample ratio of 1.5. Nucleic acids were quantified pre- and post-RNase treatment using a Thermo Scientific NanoDrop™ 8000 spectrophotometer or Qubit Broad Range DNA kit (Thermo Fisher Scientific, Waltham, MA). Several samples were processed with Maxwell LEV DNA purification kit (Promega) per the manufacturer’s guidelines and quantitated based on the same protocol as described.

### Joint sequencing of microsatellites and cancer genes

**Fig. 1:**
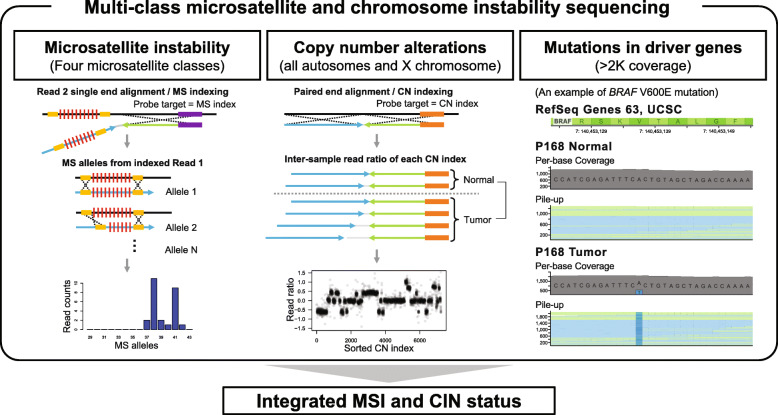
An integrated sequencing-based determination of microsatellite and chromosomal instabilities. An overview of the sequencing analysis that simultaneously determines microsatellite instability (MSI) and chromosomal instability (CIN) is shown. Mutations in driver genes also provide supplementary information that further supports the integrated determination. Shown here is an example of a BRAF V600E mutation detected from an MSI tumor

We used a targeted sequencing technology which provides ultra-deep coverage and enables amplification-free libraries with reduced PCR error (Fig. 1a) [19–21]. Referred to as oligonucleotide-selective sequencing (OS-Seq), this assay uses only a single primer, also called a primer probe that anneals to a target sequence. As a result, this method avoids issues found with traditional PCR or bait-hybridization enrichment of target exons (Additional file 1: Figure S1). Extension from the target-specific primer copies the target sequence in a massively multiplexed fashion. Primer design, library preparation, and sequencing are described in full detail in the Supplementary Methods (Additional file 1).

### Microsatellite mutation calling

We developed a bioinformatic pipeline to identify somatic changes to microsatellites [21]. Our analysis leveraged the unique property of sequencing both the targeting primer as well as the genome target. The script and required data files are available at “https://github.com/sgtc-stanford/STRSeq”. An overview of the microsatellite genotyping process is illustrated in Fig. 1a. Briefly, we used an indexing method based on the microsatellite-targeting probe sequence. After indexing based on the probe sequence in Read 2, we evaluate reads to determine whether both the expected 5′ and 3′ STR-flanking sequences are present in Read 1. This approach allowed us to identify those reads which encompassed the entire microsatellite and determine the affected allelic fraction of the microsatellite mutation. Additional description is in Additional file 1.

### Microsatellite PCR genotyping

For mononucleotide repeat instability, we used the PowerPlex MSI analysis system v1.2 (Promega) to test five markers following the manufacturer’s protocol. For EMAST, we used a set of primers that amplify tetranucleotide microsatellites (D20S82, D20S85, D8S321, D9S242, and MYCL1) as has been previously published (Additional file 2: Table S2) [13]. The 12.5-μl reaction included 2 ng of gDNA, 0.4 μM of each primer, 1X Buffer I with MgCl_2_, 0.2 mM of each dNTP, and 1.25 units of AmpliTaq Gold polymerase (Thermo Fisher Scientific). The samples were denatured at 95°C for 5 min, followed by 35 cycles of 30 s at 95°C, 90 s at 58°C, and 30 s at 72°C. The final steps for amplification involved an incubation at 72°C for 10 min and cooling to 4°C. We used ABI 3130xl Genetic Analyzer (Thermo Fisher Scientific) using a recommended matrix standard setting. PowerPlex 4C (Promega) and DS-33 (Thermo Fisher Scientific) matrix standard kits were used for the mono- and tetranucleotide repeat assays, respectively. Using the raw signal data (fsa files), the Peak Scanner v1.0 (Thermo Fisher Scientific) program provided the size of detected amplicons. We used a criterion where a shift in allele size of 3bp or more in the tumor sample compared to matching normal sample indicated instability at a given microsatellite locus.

### Identifying copy number alterations

We used the data from paired-end alignment to perform copy number analysis and somatic mutation calling. As with MSI analysis, the “ZP” sam tag is added to both R1 and R2 reads. The number of reads sharing each primer ID is determined by counting “ZP” tags and represents how many DNA molecules are captured by the primer. Therefore, by comparing the per-probe read counts between tumor and normal samples, copy number changes can be measured. The per-probe read counts were first normalized by the total number of reads from all the probes, and then the log2 ratio between tumor versus normal was calculated. When calculating the ratio, we excluded the probes that showed high variation from normal DNA as determined by a *Z*-score. Specifically, the probes having a *Z*-score greater than 2 or less than −2 were excluded. In addition, we corrected systemic biases of the log2 ratios in terms of GC% of probe capture sequence and the per-probe read counts. The adjustments were made by the locally weighted scatterplot smoothing method. For example, the values at the regression line were set to zero. Using the per-probe ratios, which were normalized and corrected, median ratios for all the target genes were calculated as a representation of copy number. These values were used for generating heat maps and also in comparison with ratios from whole genome sequencing (WGS). A focal amplification refers to a copy number gain with log2 ratio greater than 2.

### Copy number classification

We used an extreme gradient boosting algorithm called XGBoost to train a multi-class model and make predictions about the copy number classification [22]. This method was implemented in the R package xgboost. We used the copy number data from the Cancer Genome Atlas (TCGA) study for CRCs [23] as the training set. For all analysis, we used the softprob objective function and ran it 100 times. Other training parameters such as eta, gamma, minimum child weight, and maximum depth were all set as default values. An XGBoost model was then trained on the copy number cluster labels, using the 82 signature genes as features from the targeted sequencing assay.

### Identifying gene mutations and inferring clonal architecture

Paired-end read alignment was used for the detection of somatic mutations. We used the Sentieon TNseq package (v201808.03; Sentieon Inc, Mountain View, CA) to preprocess the alignments and to detect somatic variants following the best practice guidelines. Sentieon TNseq consists of tools that are based on Mutect and Mutect2 [24]. When calling mutations, we did not mark duplicates because the OS-Seq libraries are prepared without PCR amplification, i.e., a single read represents a single molecule. In addition, we masked the first 40 bases of R2 reads as N bases because they did not originate from the sample’s gDNA, but from the OS-Seq probes. We considered mutations pathogenic if they had a CADD score greater than 20 [25].

Using the full list of somatic mutations (i.e., both pathogenic and non-pathogenic), we performed a PyClone [26] analysis to infer clonal architecture of tumors with high mutation burdens with the “total_copy_number” prior. First, we estimated absolute copy numbers for each gene based on targeted or whole genome sequencing. Using the tumor purity obtained from the distribution of mutant allelic fractions, we calculated absolute copy number with an assumption of no subclonal copy number changes. For the input tsv files, we set normal_cn = 2, minor_cn = 0, and major_cn = CN_abs_, where the latter equation, CN_abs_, is the absolute copy number and also the total copy number. With the “total_copy_number” prior, PyClone considers all possible mutant copy numbers which can be equal to or less than the total copy number.

### Whole genome sequencing

To assess the copy number analysis accuracy, we sequenced matched tumor and normal samples from four patients using the Illumina MiSeq or NextSeq platform (Illumina, San Diego, CA, USA) (Additional file 2: Table S3). Sequencing libraries were prepared using 50 ng of DNA with the KAPA HyperPlus Kit as per the manufacturer’s protocol (Roche). The libraries were sequenced with a paired-end read length of 150bp. The sequence data was automatically index-assigned (i.e., normal and tumor) and aligned with BWA [27], using default parameters, against human genome build 37. Duplicate reads were removed. The data was sorted and indexed with samtools [28]. We used the program CNVkit [29] to identify copy number alterations.

We used linked read sequencing to retain long-range genomic information from three tumor/normal sample pairs **(**Additional file 2: Table S3). We prepared the sequencing libraries for the samples using the Chromium Library Kit (10X Genomics) following the manufacturer’s protocol. The library was sequenced using the Illumina NovaSeq 6000 system with 150 by 150-bp paired-end reads. The resulting BCL files were converted to fastq files using Long Ranger (v2.1.2) “mkfastq,” then Long Ranger (v2.1.2) “wgs” was run to align the reads to GRCh37.1 and detect rearrangements. We called somatic variants using the Sentieon TNseq package (v201808.03) and identified copy number alterations using CNVkit [29] following the best practice guidelines.

### Microsatellite genotyping by whole exome sequencing

For six paired tumor normal samples, we performed whole exome sequencing (Additional file 2: Table S3). Whole genome libraries were prepared using 500 ng of DNA with the KAPA HyperPlus Kit as per the manufacturer’s protocol (Roche). For a normalized pool of all the genomic libraries, exome capture was performed using xGen exome research panel v2.0 (Integrated DNA Technologies, Coralville, IA). The exome-enriched library was sequenced using the Illumina NextSeq platform with 150 by 150-bp paired-end reads. The sequence data was aligned with BWA [27], using default parameters, against human genome build 37. Using the bam files from normal and tumor samples as input, the fraction of microsatellite loci with allele shift versus the total number of measured microsatellite loci was obtained using MSIsensor-pro [30]. The unstable fraction was obtained in two individual runs using either a list of all mono- or all tetranucleotide repeats searched in human genome build 37.

### Digital PCR validation of copy number alterations

The digital PCR assays were run on a QX200 droplet digital PCR system (Bio-Rad) per the manufacturer’s guidelines. Additional details including the primers and thermocycling programs are described in Table S4 (Additional file 2) and Supplementary Methods (see Additional file 1). We assessed each patient sample with three independent replicates for gene copy number. For samples analyzed in the hydrolysis probe-based assay, droplets were clustered using QuantaSoft (version 1.2.10.0).

### Identification of chromosome arm alterations in TCGA CRC samples

TCGA CRC copy number alterations were downloaded [23]. CNTools R package was then used to convert the segment data to gene-level data. We considered a log2 ratio greater than 0.2 as a copy number gain, and less than −0.2 as a copy number loss. A chromosome arm-wide event was defined as copy number alterations among 50% or more of the genes located in a given chromosome arm and having a consistent gain or loss. For samples labeled as MSS, MSI-low (MSI-L), or MSI-H, the frequency of arm-wide events was calculated by dividing the number of altered samples by the total number of samples in the category.

## Results

### Evaluating diverse microsatellite classes and cancer drivers

We developed a cancer sequencing approach to identify somatic alterations for the major classes of tandem repeat motifs (i.e., MSI and EMAST) and to simultaneously characterize other genomic instability features, such as copy number alterations and clonal architecture (Fig. 1a). We examined 225 microsatellite markers and 1387 exons from 85 cancer genes involved in CRC biology (Additional file 2: Table S5). The microsatellite markers are from three major resources (Additional file 2: Table S6): (i) SelTarBase (http://www.seltarbase.org/) [31], (ii) driver pathway or clinically actionable genes, and (iii) traditional forensic markers (https://strbase.nist.gov/index.htm) which are known to have a significant number of germline alleles. These markers included the following: 144 mononucleotides, 37 dinucleotides, 6 trinucleotides, and 38 tetranucleotide motifs. Among the microsatellite markers with mononucleotide repeats, their motif count (i.e., number of repeats) ranged from 7 to 49. For the dinucleotide repeats, the motif count ranged from 4 to 21. For the trinucleotide repeats, the motif count ranged from 9 to 18. For the tetranucleotide repeats, the motif count ranged from 7 to 29. We included all five microsatellites that are part of the Bethesda criteria which includes mononucleotide repeats (BAT25, BAT26) and dinucleotide repeats (D2S123, D5S346, D17S250) [4]. Leveraging the results of TCGA, we identified 85 cancer genes that have among the highest frequency of mutations in CRC and are known cancer drivers [1]. These 85 genes were located across all autosomal chromosomes, as well as the X chromosome, and included *APC*, *TP53*, *KRAS*, and other well-established cancer genes (Additional file 2: Table S7) [1, 32].

### Testing microsatellite genotyping on colorectal cancers

Forty-six CRCs were used for this study (Additional file 2: Table S1). A subset of the samples had prior clinical testing for the presence of MSI. All of these samples had matched tumor and normal pairs. To improve the detection of somatic alterations that occur at lower allelic fractions, we used ultra-high sequencing coverage of microsatellite and gene targets, averaging 2,865X coverage per sample (Additional file 2: Table S8). To reduce PCR artifacts, we eliminated the PCR amplification steps for library preparation (Additional file 1: Figure S1). We developed a new analytical method for determining microsatellite mutations and MSI quantitation. For a given microsatellite locus, we calculated the distance between two samples using all of the observed microsatellite alleles (the “Methods” section, Additional file 1: Figure S2a); we determined an allele coverage proportion vector for any given microsatellite. This algorithm leveraged the improvements in sequencing data quality that resulted from reducing amplification stutter and eliminating artifactual microsatellite alleles (Additional file 1: Figure S3) [21].

**Fig. 2:**
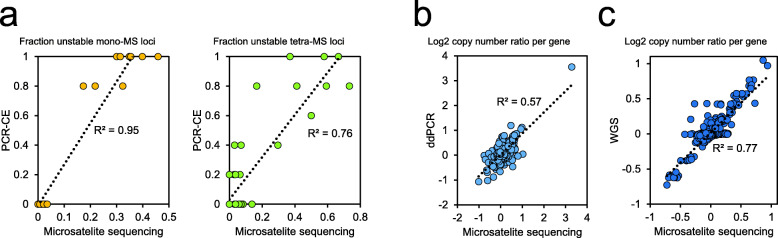
Comparison with conventional methods. **a** Comparison with PCR genotyping for mono- or tetranucleotide repeat microsatellites. For each of 46 CRCs, the fraction of unstable loci was plotted. For PCR genotyping assays, five microsatellite markers were used for each. For the sequencing assay, 144 mono- and 38 tetranucleotide repeat microsatellites were used, respectively. **b** and **c** Comparison with digital PCR **(**dPCR) and whole genome sequencing (WGS) for gene copy numbers. For each of 46 CRCs, the log2 gene copy number ratio between tumor versus matched normal samples was plotted. To validate the sequencing results, seven genes (*VEGFA*, *MET*, *FGFR1*, *CDK4*, *FLT3*, *ERBB2*, and *AURKA*) were selected for dPCR testing. For comparison with WGS, 83 target genes were used. In all the plots, dotted black lines indicate linear regression, and the correlation is indicated as an *R*-squared value

Next, we compared the sequencing-based microsatellite genotypes to the results from two PCR assays with different microsatellite panels and measured via CE. The first PCR assay tested microsatellites with mononucleotide repeats. The second assay tested those with tetranucleotide repeats that have been used to characterize EMAST. All of the samples were genotyped with both PCR assays. Overall, there was a high correlation between the targeted sequencing and PCR-based genotypes (*R*^2^ = 0.95 for mononucleotide repeats, *R*^2^ = 0.76 for tetranucleotide repeats; Fig. 2a). This result indicates that the sequencing-based microsatellite genotypes were generally accurate for mono- and tetranucleotide repeats.

Finally, we compared our method to whole exome sequencing (Additional file 2: Table S9). Between the two targeted sequencing methods, we observed a high correlation for the instability levels of both mono- and tetranucleotide repeats (*R*^2^ = 0.99 for mononucleotide repeats, *R*^2^ = 0.88 for tetranucleotide repeats). However, conventional exome sequencing had limited sensitivity because the tetranucleotide repeats targeted by the whole exome capture were mostly short repeats (Additional file 1: Figure S4). There was a false-positive case (P6261); exome sequencing detected no allelic shifts in tetranucleotide repeats while our method detected allelic shifts in 16.7% of the targets.

### Detection of somatic copy number alterations

To detect copy number events that co-occur with MSI, we developed a new method to accurately measure copy number alterations (the “Methods” section; Fig. 1a). This feature leveraged the highly reproducible targeting performance of this approach [19]. For any given DNA sample, the number of sequencing reads generated from an individual probe was highly reproducible across replicates and different DNA samples (Additional file 1: Figure S5). Thus, based on the read ratio of tumor versus normal samples after normalization (the “Methods” section), a copy number was determined for each gene.

To validate the accuracy, we compared the copy number measurements to the results from two independent methods. We tested a subset of samples with digital PCR copy number assays for six target genes (Fig. 2b). This comparison showed a high correlation between the MSI sequencing assay and digital PCR (*R*^2^ = 0.59), supporting the accuracy of the current approach for copy number determination. In addition, we conducted WGS studies of seven sample pairs (Additional file 2: Table S3**)**, and the targeted gene copy number changes were strongly correlated with the WGS results (*R*^2^ = 0.77; Fig. 2c).

### Profiling CRC microsatellite instability across different sequence repeat motifs

We determined the extent of MSI across different categories of tandem repeats (Additional file 2: Table S10). Among the 225 microsatellites that were sequenced, 129 of them had a somatic mutation leading to an allelic shift as detected in at least one CRC (Additional file 2: Table S11). A subset did not have any somatic mutations or allelic shifts; they were characterized by short repeat lengths and included mono- and dinucleotide repeats up to 10 bp in length. In other words, all mono- and dinucleotide repeats longer than 10 bp had a mutation among the 46 CRCs. Nearly all of the other tri- and tetranucleotide repeat microsatellites (*N* = 44) had at least one microsatellite mutation across the entire set of CRCs. The only exception involved the tetranucleotide D4S2364, which had no mutations. Interestingly, the microsatellite mutation fractions in mono- and tetranucleotide repeats demonstrated a high correlation (*R*^2^ = 0.90; Additional file 1: Figure S6), meaning that mutations in mono- versus tetranucleotide repeats were associated with one another.

**Fig. 3:**
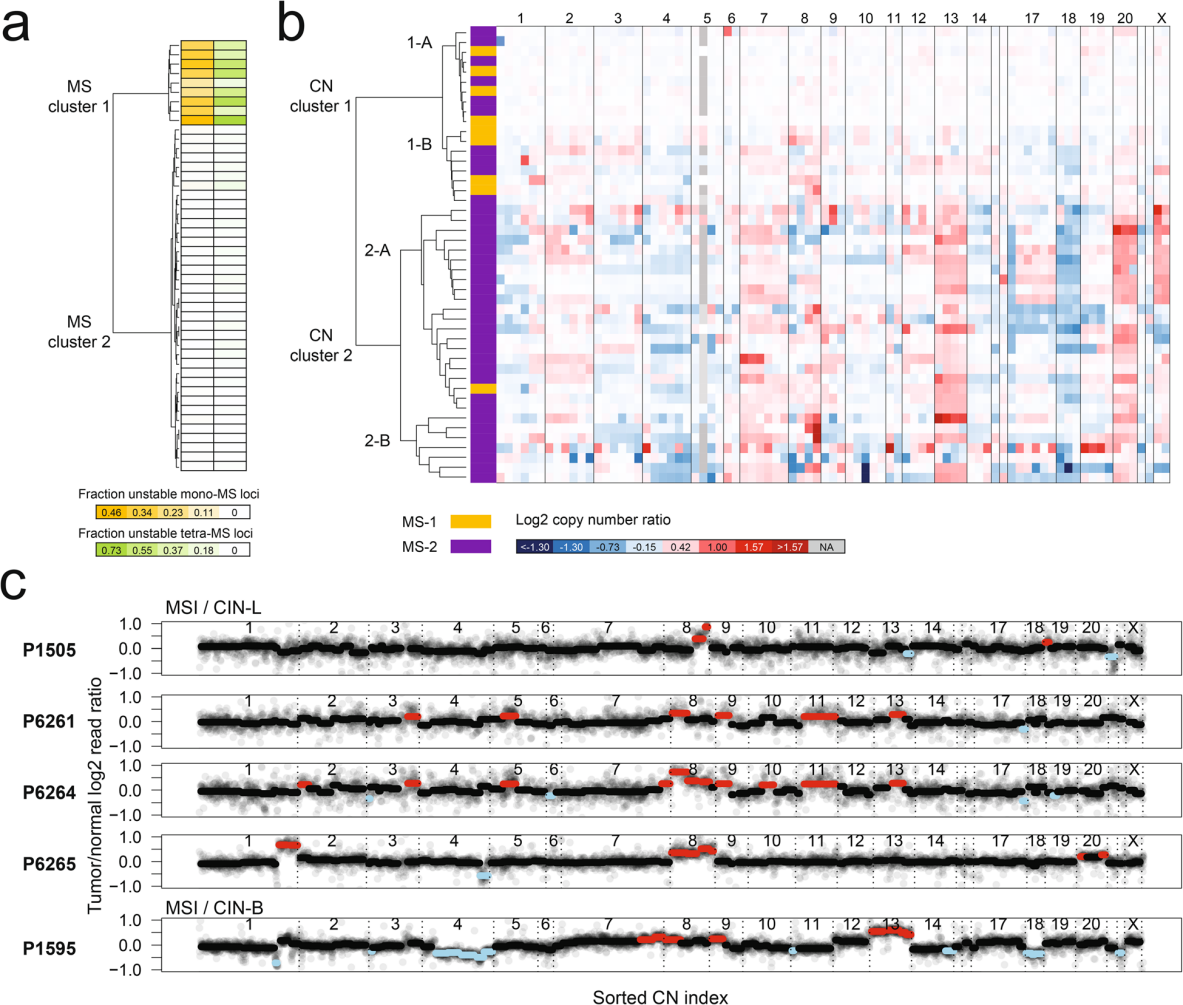
Profiling diverse sequence tandem repeats and gene copy numbers in 46 colorectal cancers. **a** Clustering based on 225 microsatellites across four different classes. A 225 × 46 matrix including the presence (1) or absence (0) of microsatellite allele shift mutations was used for an unsupervised hierarchical clustering, which generated two clusters (MS Clusters 1 and 2). The heatmap of the two microsatellite classes (mono- and tetranucleotide repeats) with the most contributions are shown in two separate columns. **b** Clustering based on tumor/normal copy number ratio of 83 target genes. The median log2 ratios for all the target genes were used for an unsupervised hierarchical clustering, which generated two major clusters. Each major cluster has two subclusters. In the first column of the heat map, the MS Cluster identification for each CRC is indicated as a different color. The numbers on top of the heatmap indicate the chromosome where the genes are located. Copy number gain and loss are indicated with red and blue colors, respectively. **c** Log2 copy number ratio plots for all the CRCs having both MSI and CIN. For each CN index (*x*-axis), the log2 copy number ratio between read counts from tumor and normal samples (*y*-axis) is plotted. The median ratio value is indicated with lines of black, red, or sky blue, representing no change, copy number gain, or loss, respectively

Next, we conducted an unsupervised analysis based on the microsatellite mutation profiles. As shown in Fig. 3a, the hierarchical clustering extrapolated from microsatellite (MS) mutations identified two major groups: MS Cluster 1 (*N* = 9) and MS Cluster 2 (*N* = 37). All of the CRC samples in MS Cluster 1 had a higher percentage of unstable microsatellite loci across all classes of microsatellites than MS Cluster 2 (Fig. 3a; Additional file 1: Figure S7). Overall, the mean fraction of unstable microsatellite loci was 29.0% for Cluster 1 versus 1.2% for Cluster 2 which was statistically significant (*p* < 0.001). MS Cluster 1 CRCs had all the features indicative of both MSI and EMAST.

### Comparison with conventional detection for MSI-H and EMAST

From the same CRC samples, we used PCR and IHC assays to determine the MSI-H and EMAST status (the “Methods” section). We used an MSI PCR panel (NR-21, NR-24, BAT25, BAT26, and MONO-27) with the Bethesda criteria defining MSI-H status requires two or more microsatellites to show size shifts based on somatic alterations. CRCs with only one microsatellite with an allelic shift indicate MSI-L. Nine CRCs were MSI-H according to the PCR testing results (Additional file 2: Table S12). Eight of these CRCs from MS Cluster 1 had also undergone clinical IHC testing for the four MMR proteins (MLH1, MSH2, MSH6, and PMS2) (Additional file 2: Table S1). All eight of these samples lacked MMR protein expression, which was consistent with the MSI status determined from the sequencing results. Then, we tested a set of five tetranucleotide repeats (D20S82, D20S85, D8S321, D9S242, and MYCL1) previously used to determine EMAST status [13]. If two or more show a size shift, this indicates EMAST instability. Eleven CRCs were classified as EMAST positive (Additional file 2: Table S13). Finally, no tumors had evidence of MSI-L per PCR testing.

Based on comparing NGS to PCR testing, we concluded that MS Clusters 1 and 2 were indicative of MSI and MSS status, respectively. Among this set of samples, none of the CRCs had MSI-L. In addition, we did not identify any CRCs that were exclusive to EMAST or MSI-H, i.e., MSI globally affects all classes of microsatellites. All nine tumors in MS Cluster 1 were positive for both MSI-H and EMAST in the PCR tests. Again, this validation result suggests a general association of MSI-H and EMAST. All of the tumors in MS cluster 2 were negative for MSI-H per PCR testing (Additional file 2: Table S12). This result was generally corroborated by the sequencing results where only a small fraction of mononucleotide markers had somatic mutations. Overall, these results confirmed that our sequencing method was fully concordant with MSI PCR.

For determining EMAST, NGS and PCR tests were discordant for a small number of samples. Two of 37 CRCs (P544 and P685) in MS Cluster 2 were EMAST positive as denoted by somatic allelic shifts in two markers based on PCR analysis with CE. Per the microsatellite sequencing analysis, the P544 CRC had 7.1% of the tetranucleotide markers with mutations. The P685 tumor had an even lower frequency at 3.1%. The mutation frequency among tetranucleotide markers was significantly lower in MS Cluster 2 compared to MS Cluster 1 (2.9–13.8% versus 16.7–73.3%, *p* < 0.001). The two CRCs only positive for EMAST by PCR test are likely to be false positives.

### The 3-bp shift criterion improves MSI classification

We determined that microsatellite markers varied in their accuracy for detecting MSI. From the sequencing results, we examined reads covering the microsatellite markers used in the Bethesda panel and three overlapping markers provided in a commercial set (Promega) (Table 1). Our sequencing analysis detects any size of somatic indel shift compared to the matched normal tissue genotype, even as small as 1 bp (Additional file 1: Figure S3). Especially for mononucleotide repeat markers in PCR assays, a 3-bp shift cutoff minimizes false-positive detection due to PCR assay variation [33]. However, the results from the current study suggest the criterion should also be used for dinucleotide repeat markers. With the 3-bp shift criterion applied to both mono- and dinucleotides markers, the accuracy of the Bethesda panel improved (Table 1).

**Table 1:** Frequency of allele shifts in traditional MSI markers

			All shifts			≥3bp shifts		
STR ID	Panel	Motif	Frequency in MSI (*N* = 9)	Frequency in MSS (*N* = 37)	Accuracy	Frequency in MSI (*N* = 9)	Frequency in MSS (*N* = 37)	Accuracy
NR-21	Powerplex	A	100%	0%	100%	100%	0%	100%
BAT26	Bethesda, Powerplex	A	100%	19%	84%	100%	0%	100%
BAT25	Bethesda, Powerplex	T	100%	28%	76%	100%	0%	100%
D17S250	Bethesda	GT	100%	29%	74%	67%	0%	97%
D5S346	Bethesda	TG	78%	0%	96%	22%	0%	85%
D2S123	Bethesda	CA	67%	0%	93%	56%	0%	91%
**MSI-H with all Bethesda markers**^a^			100%	19%	85%	100%	0%	100%

^a^Two or more shifts among five markers determine MSI-H

Notably, indel shifts of 3 bp or greater in length occurred only in MSI tumors (Additional file 2: Table S14). In contrast, MSS tumors did have low levels of 1- or 2-bp allelic shifts, particularly for mono- and dinucleotide microsatellites. Specifically, there were 27 MSS tumors with the microsatellite indel shifts smaller than 3 bp in size: 12 had at least two microsatellites that were affected; the remaining 15 had only a single affected microsatellite.

### Analysis and classification of copy number alterations

Similar to the analysis of MSI, we conducted a separate unsupervised clustering using only the copy number (CN) alterations from 83 targeted genes with the most reproducible copy number calling results. The analysis identified two major CN clusters (Fig. 3b). CN Cluster 1 had a total of 18 CRCs and on average only 7% of genes had a CN alteration. CN Cluster 2 had the remaining 28 CRCs which had a significantly higher number of CN alterations, affecting 44% of the genes on average. The differences in CN between the two clusters were highly significant (*p* < 0.001, Additional file 1: Figure S8a).

CN Clusters 1 and 2 had distinct subclusters (Fig. 3b). CN Cluster 1 had two subclusters (Additional file 1: Figure S8b). The first cluster, CN Cluster 1-A (*N* = 10), had copy number changes averaging less than 1% of the genes per sample, which is in line with a chromosomally stable (CS) state. The second cluster, CN Cluster 1-B (*N* = 8), had copy number changes evident in the range of 6–26% of the genes across these samples, demonstrative of a lower level of CIN. This difference between the two subclusters was highly significant (*p* < 0.001).

Likewise, CN Cluster 2 had two distinct subclusters. CN Cluster 2-B (*N* = 7) had high-amplitude focal gene amplifications with six or more copies per gene or the presence of homozygous deletions. CN Cluster 2-A (*N* = 21) had copy number changes of broader genome segments that could extend over entire chromosome arms. A focal amplification of *MYC*, as denoted by a log2 ratio greater than 2, was present in the P98 and P685 CRCs. *MYC* is a well-known oncogenic driver associated with amplifications. The tumor suppressor gene, *TP53*, was one of the most frequently deleted genes (*N* = 21), as has been observed among other studies [34]. In addition, we identified a series of chromosome-wide events (i.e., copy number gain or loss of all the target genes across the arm of a given chromosome). For example, chromosome-wide copy number gains of Chromosome 13 (*N* = 16) and Chromosome 20 (*N* = 17) and loss of Chromosome 18 (*N* = 21) were the most frequent among all of the CRCs.

### Validating the determination of the chromosomal instability classes

To validate the detection of different categories of CIN, we used a multi-class statistical model based on the copy number alterations for the targeted 83 cancer genes on an independent data set. For training, we used copy number information from the TCGA CRC copy number dataset (*N* = 339) based on genome-wide analysis with arrays. Liu et al. reported that colorectal cancer had specific classes of CIN involving either focal (CIN-F) versus broad (CIN-B) genomic copy number changes [35]. A statistical threshold was used to define the two major classes. CIN-F was characterized by high-amplitude focal amplifications whereas CIN-B had low-amplitude copy number gains that spanned broader segments of the genome.

For the current study, we trained the 83 gene classifier using TCGA CRC results from the study of Liu et al. [35]. Then, we determined how the hierarchical CIN clustering (Clusters 1 and 2) overlapped with the CIN states (i.e., CS, CIN-F, and CIN-B) defined by Liu et al. The sensitivity and accuracy of the model were evaluated by performing fivefold cross-validation. The model had an overall prediction accuracy of 100% (sensitivity 1, specificity 1), indicating that the CIN cluster results overlapped precisely with the CIN-B and CIN-F states based on the TCGA data set. Additionally, when we reversed our training data and validation TCGA datasets, we found the same level of sensitivity and specificity. Thus, we concluded that CN Cluster 2A indicated CIN-B while CN Cluster 2B indicated CIN-F.

In terms of CIN Cluster 1, we observed a distinct subset. Cluster 1-B had a significantly higher number of affected genes than Cluster 1-A (Additional file 1: Figure S8b). In addition, the amplitude of copy number changes in Cluster 1-B was significantly higher than that in Cluster 1-A; the difference was measured by comparing the variances of log2 gene copy number ratios (*p* < 0.001, Additional file 1: Figure S8c). Given this significant difference, we classified CRCs in CN Cluster 1-B as chromosome instability low (CIN-L), an indicator of the low degree of copy number changes. The remaining CRCs were considered to be CS.

### Profiling MSI and CIN co-occurrence in CRCs

From the current sample set, we determined if the sequencing approach could identify the co-occurrence of these genomic instability states. Among the nine CRCs with MSI, five had evidence of co-occurring copy number alterations and, thus, indicators of CIN. Four CRCs (P1505, P6261, P6264, and P6265) had both MSI and CIN-L (Fig. 3c). Interestingly, all four tumors had copy number gains among four genes (*WRN*, *FGFR1*, *TRPS1*, and *MYC*) which are located on Chromosome 8. No copy number losses were noted among these genes.

In contrast, MSS CRCs had both copy number gains and losses among these same Chromosome 8 genes. Specifically, there were 31 MSS tumors with CIN-L, CIN-B, or CIN-F. Nineteen of these tumors had at least one copy number loss among the four genes on Chromosome 8. Notably, the majority of the losses included genes on the 8p arm; fourteen of the MSS tumors had no losses of the genes located on the 8q arm, but only of the genes on the 8p arm.

One tumor had a striking and distinct pattern of mixed genomic instability. The P1595 CRC was MSI/CIN-B. This tumor had the highest number of chromosome-wide copy number changes among the MSI CRCs (Fig. 3c). We detected copy number gains among the genes in Chromosomes 8 and 13 as well as losses in Chromosomes 4 and 18. This pattern matched that of the CRCs which were MSS and CIN positive. This combination of genomic instability features would have been missed with conventional MSI PCR testing.

### Using a deep sequencing approach to identify DNA repair mutations

**Fig. 4:**
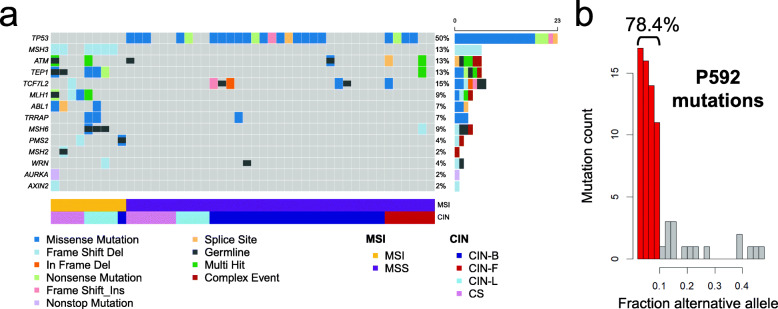
Mutation profile for genes related to DNA maintenance. **a** Oncoplot for the DNA maintenance genes. For all of the genes related to DNA maintenance (rows), mutation profiles of 46 tumors (columns) are shown. Only the mutations with a CADD score greater than 20 are used. Different types of somatic mutations are shown as rectangles with different colors. Gray color indicates that there is no mutation call at a given gene. Germline mutations are also indicated with a shorter rectangle overlaid on the somatic mutation map. The right panel shows the number of affected samples for each gene. Genes are sorted according to the frequency of somatic mutations. The lower panel indicates MSI and CIN sample annotations determined by the sequencing assay. **b** Distribution of alternative allelic fractions in the P592 tumor. The mutations with an allelic fraction less than 0.1 are indicated with red color, and the percentage is also provided on top of the corresponding histogram bars

From the deep sequencing of the cancer genes, we identified mutations including substitutions and indels among well-established CRC driver genes (Additional file 1: Figure S9; Additional file 2: Table S15). As one would expect, the MMR genes (e.g., *MLH1*, *MSH2*, *MSH3*, *MSH6*, and *PMS2*) had different mutation frequencies when comparing the MSI versus MSS CRCs (Fig. 4a). All nine of the MSI CRCs had at least one somatic mutation in an MMR gene. Six of the MSI CRCs had germline mutations of MMR genes and four of them also had a somatic mutation in the gene with a germline hit. The only MSS tumor with an MMR mutation was P592, which had a somatic mutation in *MSH6* as we describe in more detail later.

We examined the 16 genes which play a role in DNA repair and genome stability (Additional file 2: Table S7). Our results included the following: 10 genes had frequent mutations among the MSI tumors, one gene had mutations among the MSS tumors, and three genes had mutations in both MSI and MSS tumors. Notably, MSI tumors had no mutations in *TP53* versus 62.2% of MSS CRCs that had *TP53* mutations.

An *MSH3* indel was found to be a hotspot mutation among the MSI tumors (66.7% in MSI versus none in MSS). This recurrent indel was at an adenine mononucleotide repeat, located at exon 7 of *MSH3*; this mononucleotide homopolymer is described as being eight bases in the genome reference. Among the five CRCs with MSI and CIN, *MSH3* mutations were detected in four tumors. For two of them (P1505 and P6264), the mutant allele fraction was as high as the tumor purity, suggesting biallelic mutation. Interestingly, the two CRCs with biallelic *MSH3* mutations had higher fractions of unstable tetranucleotide loci than all the other MSI tumors. *MSH3* mutations did not co-occur with *TP53* mutations. A similar level of exclusivity was evident among other sets of CRCs including those analyzed in the TCGA study, where among 323 CRCs with a mutation in either gene, 95% were exclusive to one or the other [36].

A notable example of mixed genomic instability states was evident in the P592 tumor. This CRC was MSS per the sequencing analysis and the MSI PCR test. However, this CRC had 74 mutations, which was even higher than the average mutation count of MSI CRCs (43 mutations) that are normally hypermutable **(**Additional file 2: Table S16). We observed that 78.4% of P592 CRC’s mutations occurred at a lower allelic fraction of 10% or less. This lower mutation allele fraction represented a subclonal population of tumor cells (Fig. 4b). Both our ultra-deep sequencing and whole exome sequencing detected no somatic mutations in both *POLE* and *POLD1* genes. Interestingly, we discovered a mutation in *MSH6* that was present at a somatic allelic fraction of 6.3% and led to a frameshift. Among all of the MSS tumors, this CRC was the only one with a mutation in an MMR gene. We also noted a copy number loss per a log2 ratio of −0.11, a lower value that we attribute to the deletion being in a small proportion of tumor cells (Additional file 4). Moreover, this was one of the MSS tumors that had many small somatic shifts (i.e., 1 or 2 bp) in mono- and dinucleotide microsatellites as determined by deep sequencing (Additional file 2: Table S14).

### Ascertaining the subclonal structure of CRCs with mixed genomic instability

**Fig. 5:**
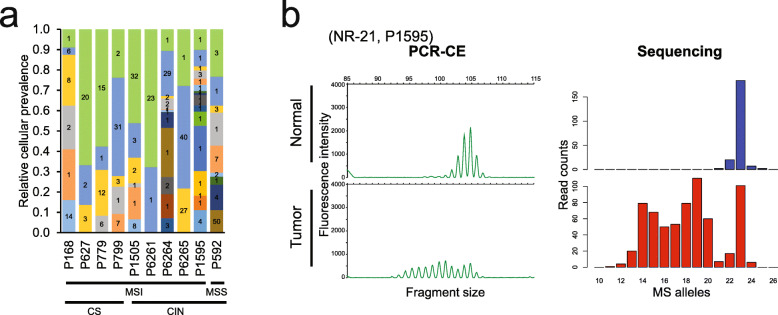
Clonal diversity of hypermutated tumors. **a** Clonal diversity analysis for hypermutated tumors. Relative cellular prevalence of each clone, indicated by overlapping bars in the plot, was estimated by PyClone based on the allelic fraction of the mutations shared by each clonal population. The number of mutations for each clone is indicated on the inside of each bar. **b** Clonal diversity shown in MSI analysis. For a microsatellite locus (NR-21), allele profiles of both normal and tumor samples from P1595 are shown. Electropherograms generated by the PCR-CE method (left panels) and the allele histograms generated by our deep sequencing approach (right panels) are compared. For both panels, *x*- and *y*-axes indicate individual microsatellite alleles and their relative abundance, respectively. From the tumor allele profile, many different allelic shifts are observed

We determined the subclonal structure and the relative population sizes among the ten CRCs with hypermutations (Fig. 5a; Additional file 1: Figure S10; Additional file 2: Table S17). Our results revealed a diverse range of tumor cellular architecture types across MSI CRCs. For this analysis, we used PyClone; this algorithm relies on targeted sequencing data with high coverage, deconvolutes the clonal architecture of tumors, and estimates the subclonal cellular prevalence of somatic mutations [26]. Subclonal analysis can be limited by the relatively small number of mutations from targeted sequencing data. For this study, we focused on the overall subclonal structure, not individual subclones determined by the analysis.

For any given hypermutated tumor, we observed different levels of subclonal diversity, which can be inferred by the number of mutation clusters. All ten CRCs had two or more clones as defined by groups/clusters of mutations with similar degrees of cellular representation (Additional file 2: Table S17). Among the CRC mutations that were pathogenic (i.e., variants with a CADD score greater than 20), we noted specific patterns in terms of their clonal clustering distribution. Deleterious mutations generally occurred in larger sizes than the clusters without pathogenic variants (mean values 1.2 versus 8.6 variants per cluster, *p* < 0.001) (Additional file 1: Figure S11). A range of subclonal heterogeneity was observed across the MSI tumors with CIN. For example, the P6261 CRC (MSI/CIN-L) had only two mutation clusters; the cluster with the largest size included 96% of the mutations.

The P1595 CRC (MSI/CIN-B) had 18 distinct mutation clusters, where the largest cluster contained only 17% of the mutations. Most of the clusters were defined by only one mutation, and therefore, some of the subclone distinctions may be incorrect. However, high clonal diversity was apparent considering the mutations in such clusters being the majority (70% of all somatic mutations). This result was validated when comparing to a separate exome-based analysis of the same tumor, where 41% of somatic mutations were in single-mutant clusters (Additional file 2: Table S18). Among the MSI tumors, this CRC had the highest level of clonal diversity as well as the highest number of copy number alterations. The high clonal diversity of P1595 CRC was also confirmed by a diverse range of tumor microsatellite mutations (Fig. 5b). For example, a pentanucleotide repeat marker showed six different alleles in P1595 CRC (Additional file 1: Figure S12a). In contrast, the P799 CRC, with less clonal diversity, did not have as broad a range of tumor microsatellite alleles (Additional file 1: Figure S12b). Notably, this tumor had both *APC* and *BRAF* mutations, which are generally exclusive [37], identified in separate subclones based on their prevalence (Additional file 2: Table S17). The *APC* mutation was in a mutation cluster with a cellular prevalence of 0.59, while the *BRAF* mutation was in a mutation cluster with a cellular prevalence of 0.11.

With the predicted cellular prevalence for each somatic mutation, we could also determine if a mutation was biallelic (Additional file 2: Table S19). Nine biallelic pathogenic mutations were identified, of which the adjusted allelic fraction among the mutant population was 80% or more. We determined biallelic mutations only from the subclonal population represented by more than ten somatic mutations.

### Validation with a comparison to whole genome analysis

**Fig. 6:**
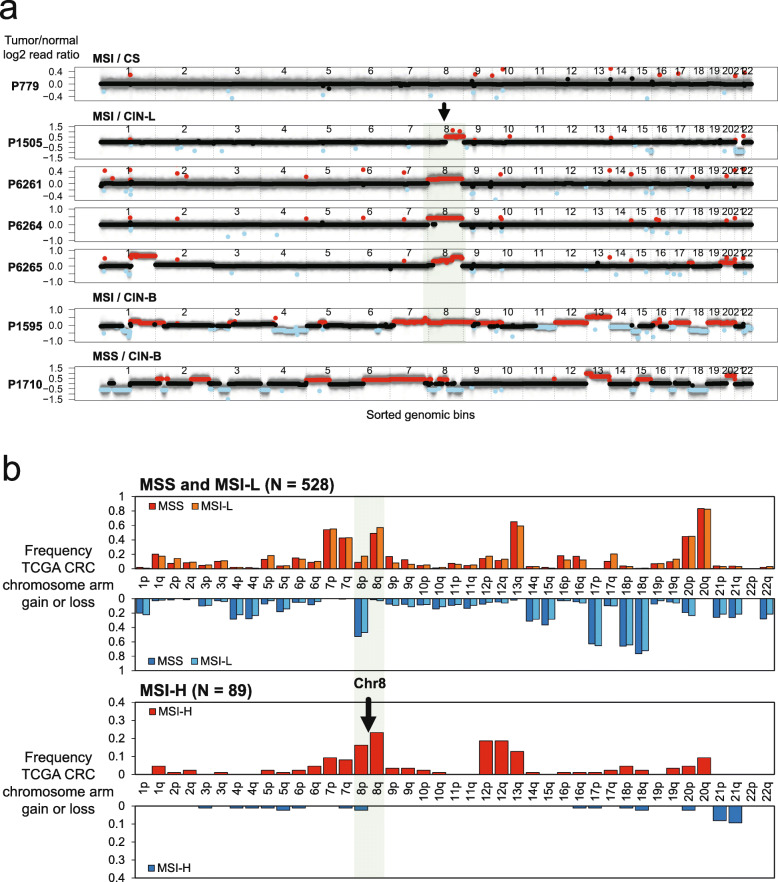
Genome-wide copy number changes in MSI tumors determined by WGS analyses. **a** Log2 copy number ratio plots from the WGS analysis. This analysis included all five MSI/CIN samples, as well as one MSI/CS and one MSS/CIN sample as controls. For each genomic bin (*x*-axis), the log2 copy number ratio calculated by CNVkit (*y*-axis) is plotted. The log2 ratio value of genomic segments is indicated with lines of black, red, or sky blue, representing no change, copy number gain, or loss, respectively. The dotted vertical lines separate genomic bins from different chromosomes. **b** Validation of chromosome arm-wide copy number events in MSI tumors using TCGA CRC samples (*N* = 617). Separately for MSS/MSI-L and MSI-H tumors, frequencies of copy number gain and loss are shown for each chromosome arm. Gains are shown above and losses below the labels of chromosome arms

To validate the results on the genomic instability status with this targeted assay, we applied WGS to five CRCs (P1505, P6261, P6264, P6265, and P1595) with mixed genomic instability features and two CRCs (P779 and P1710) with only one class of genomic instability (Fig. 6a; Additional file 2: Table S3). Congruent with the targeted sequencing analysis, the WGS results confirmed the presence of notable levels of CIN in primary MSI CRCs.

Specifically, the P779 CRC was MSI/CS and the P1710 CRC was MSS/CIN. To improve the detection of rearrangements, we used linked read sequencing on a subset of samples (P779, P1505, and P1595). The samples had high-quality HMW DNA molecules, typically in a size range of 20–40 kb on average. The HMW DNA provided phased haplotype blocks of 0.5–4.6 Mb in size (Additional file 2: Table S3).

We compared the WGS and targeted sequencing calls for copy number alterations (Additional file 2: Table S20). We considered a genome-wide metric involving the fraction of segments with a copy number change over the entire breadth of the genome. Comparing the two results demonstrated concordance across all samples. Our targeted sequencing classification of CIN exactly matched the WGS results (Additional file 2: Table S20). The CIN-B tumors (*N* = 2) had at least 37% or greater of their genome covered by copy number alterations. The CIN-L tumors (*N* = 4) had 5% or greater of their genome affected by copy number changes. Importantly, the CRCs with CIN-L (P1505, P6261, P6264, P6265) had a consistently higher degree of genomic instability than the P779 CRC with MSI/CS which was consistently diploid in its profile.

CIN events included increased copy number changes that encompassed either one or both arms of a chromosome, the latter being an example of aneuploidy. Such broad genomic copy number changes were observed in all of the MSI/CIN-L tumors—this whole genome result confirmed what we observed in the targeted sequencing analysis (Fig. 6a). For example, the P6265 CRC (MSI/CIN-L) had a copy number increase in chromosome arms 1q, 8q, and both arms of 20, indicating aneuploidy of this chromosome. P1505 had a copy number increase in chromosome arm 8q and a loss of both arms of chromosome 21.

Among the CRCs analyzed with whole genome evaluation, we detected an increased copy number of Chromosome 8, which corroborated our results from deep sequencing analysis. To validate the observation among a larger number of tumors, we examined the TCGA CRCs (*N* = 617) and their genomic copy number data [23]. Based on the CN profiles, we determined the status of chromosome arm copy number (the “Methods” section). The 8q gain was very common among MSI-H tumors (*N* = 89) with 23% (*N* = 21) having evidence of this event (Fig. 6b). In addition, 16.3% of TCGA MSI CRCs had an increase in the 8p arm copy number. Likewise, MSI CRCs had frequent copy number alterations affecting Chromosomes 7, 12, 13, and 20. Other studies corroborated this finding that 26 to 61% of MSI tumors have copy number alterations or features of CIN (Additional file 2: Table S21) [38–45].

**Fig. 7:**
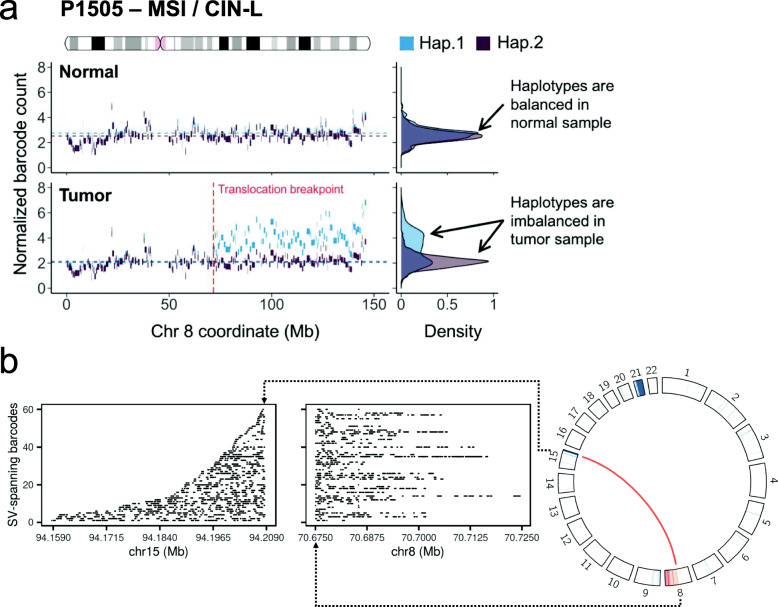
Inter-chromosomal rearrangement in a CRC tumor with a mixed MSI/CIN phenotype. **a** Haplotypes of chromosome 8 in the normal and tumor samples of P1505. The blocks indicate the original haplotype blocks determined by linked read sequencing, and their color denotes their parental assignment. Haplotype 1 (blue) in the tumor sample displays an allelic imbalance on chromosome 8q that reflects a duplication event, beginning at the translocation breakpoint with chromosome 15. The density plots to the right reflect the distribution of the haplotype counts. **b** The translocation event from the tumor sample of P1505 (an MSI/CIN-L tumor). For the CIRCOS plot (right), an inter-chromosomal change is indicated with an orange line. Chromosomes are indicated as curved boxes along the circle, with chromosome 1 at the 12 o’clock position, and continuing in a clockwise direction. The width of the box represents the size of the chromosome. Inside the boxes, the log2 copy number ratio from genomic segments is displayed as a heatmap, where orange and blue colors indicate copy number gain and loss, respectively. The left panels show molecular barcodes from linked read sequences that are found in two genomic regions flanking the breakpoints of the translocation. Each row indicates individual DNA molecules. The alignment of barcoded reads is indicated by horizontal lines located at the genomic positions (*x*-axis)

With linked read WGS, we discovered an inter-chromosomal rearrangement event in the P1505 CRC (MSI/CIN-L). Using linked read sequence data, we performed digital karyotyping [46] to identify chromosome arm alterations that are assigned a specific haplotype. This analysis produces information similar to conventional karyotyping or fluorescent in situ hybridization (FISH) but also has the advantage of having the resolution of WGS. After conducting this haplotype analysis, it became clear that a specific haplotype of 8q had been duplicated beginning at a specific breakpoint in the q arm proximal to the centromere (Fig. 7a). On closer analysis of this breakpoint, we identified a novel translocation between Chromosomes 8 and 15 that has never been reported in colon cancer (Fig. 7b). The breakpoints were located at 8q13.3 and 15q26.2, and the Chromosome 8 breakpoint separates exons 2 and 3 of *XKR9*. Overall, our approach identified MSI CRC where Chromosome 8 had a predilection for CIN.

### Lower limit of coverage for accurate genomic instability quantification

With relatively high coverage, we have analyzed the genomic instability features of CRCs. To determine the lower limit of coverage for accurate genomic instability quantification, we downsampled the sequence data from a CRC pair (normal and tumor samples from P1595). For MSI quantification, approximately 30X coverage was enough for both MSI and EMAST (Additional file 1: Figure S13). When target coverage was between 10X and 30X, only MSI (i.e., instability in mononucleotide repeats) was able to be quantitatively determined—EMAST could not be determined. When the target coverage was less than 10X, the quantitative precision for MSI was compromised and had a lower detection rate. For gene copy number quantification, all the dilutions down to 50X coverage had a high correlation with the WGS-based result (*R*-squared value of 0.9 or higher) (Additional file 1: Figure S14).

## Discussion

We developed an ultra-deep sequencing approach that enabled accurate analysis of different genomic instability states that include tetranucleotide repeats and clonal features [19, 20]. Our approach was validated in a pilot study on a set of primary CRCs. Using the new method, MSI tumors showed distinct characteristics: (i) The MSI tumors had varying degrees of microsatellite mutation with both mono- and dinucleotide repeats being proportionally elevated along with tri- and tetranucleotide repeat alterations, (ii) some MSI tumors showed CIN with chromosome- or chromosome arm-wide copy number changes as well as a translocation, and (iii) the simultaneous MSI profiling across a larger number of microsatellites and clonal architecture deconvolution revealed examples of MSI subclones coexisting with other clonal populations. This added feature of intratumoral heterogeneity may contribute to different tumor phenotypes.

Traditional PCR tests sometimes lead to a classification of “low” status, generally defined as positive only in a portion of markers such as what is observed for MSI-L and EMAST-L. Like the traditional MSI tests, PCR tests for EMAST tumors also use as few as five markers [13–15, 47]. Some recent studies used a commercial assay using 16 forensic markers [16], but the expanded number led to a new designation of EMAST-L tumors, i.e., inconclusive between positive and negative. In the current study, with an expanded number of tetranucleotide markers, we obtained definitive results about the extent of EMAST and its association with MSI and did not identify this EMAST-L category. The results suggest that a wider range and greater number of tetranucleotide markers are important for the accurate determination of EMAST.

MSI tumors had a range of different MSI fractions (Additional file 2: Table S10). Although with a limited resolution, the MSI PCR test using five mononucleotide markers generated results matching the microsatellite mutation fraction measured by our sequencing analysis (*R*^2^ = 0.95; Fig. 2a). A recent study about genetic heterogeneity of MSI tumors revealed that the overall genomic MSI level, termed “MSI intensity” in the study, is a predictor of response to immunotherapies [48]. Given the clinical implication, an improved MSI test would go beyond a simple positive versus negative indication, but would measure the quantitative extent of MSI.

We separated MSI markers into two groups according to their repeat motif length (mono- and dinucleotide repeats versus tri- and tetranucleotide repeats), and then compared microsatellite mutation rates within each group. There was a clear correlation between the length of microsatellites and the mutation frequency across all tumors regardless of their MSI status (Additional file 1: Figure S15). This result shows that many MSI markers are not specific, especially when they are long. All the traditional microsatellite markers are relatively long (>20 bp) and some of them were frequently mutated even in MSS tumors (Table 1 and Additional file 2: Table S11). To be both sensitive and specific in MSI detection, any molecular test should include more markers with enhanced specificity (e.g., markers with intermediate length).

When too short, on the other hand, a microsatellite marker generally suffers from limited sensitivity. Using publicly available WGS data from 11 CRCs with MSI [49], we investigated the sensitivity of mononucleotide repeat markers with respect to their length (Additional file 1: Figure S16). Mononucleotide repeats of 9 bp or longer had a reasonably good sensitivity for MSI cancers (>10%). The length of repeat positively correlated with the sensitivity when the repeat length is shorter than 20bp. When longer than 20bp, the sensitivity decreased most likely due to limited coverage of reads that span the entire repeat.

Another noteworthy feature of MSI tumors was that the microsatellite mutation fractions in mono- and tetranucleotide repeats were highly correlated (*R*^2^ = 0.90; Additional file 1: Figure S6). Therefore, all the MSI tumors unstable in mono- and dinucleotide repeats were also unstable in tri- and tetranucleotide repeats which define the EMAST molecular phenotype. We did not identify CRCs with instability that were exclusive to mono- and dinucleotide repeats or tri- and tetranucleotide repeats. Most studies based on PCR tests reported tumors with only a single type of instability (i.e., MSI-H only or EMAST positive only), which were as frequent as the tumors having both types of instability [13–16, 47]. However, a very recent study based on WGS found no evidence of the EMAST-only phenotype among 248 CRCs [49], which corroborated our results. In summary, the MSI tumors identified by PCR tests may not characterize the full diversity of microsatellite instability across different motif repeats.

It is thought that a major driver of EMAST in MSS tumors is *MSH3* loss-of-function [12]. The current study points to the possibility that MSI-H tumors may obtain the EMAST phenotype as a result of the initial instability in mononucleotide repeats. Other studies of CRC have reported *MSH3* mutations, but given their reliance on lower coverage methods (in the hundreds at most) such as exome sequencing, they lacked the sensitivity to detect the hotspot indel that we identified.

Generally, it is thought that dysfunction among different DNA repair mechanisms leads to exclusive states of genomic instability, such as MSI or CIN [50]. In the current study, the majority of MSI tumors had both EMAST and CIN features (four CIN-L and a CIN-B), indicating a mixed genomic instability state. Interestingly, all of the MSI/CIN-L tumors had a frameshift mutation in *MSH3*, which also supported the new classification. Changes in MSH3 function may lead to double-strand breaks and chromosomal rearrangements [51]. Overall, these unexpected structural rearrangements in MSI tumors suggest the presence of genomic heterogeneity of CRC tumors. Therefore, to be more precise in assessing the genomic properties of MSI tumors, we would recommend that determining the CIN status should be a supplementary biomarker. The method described in this study enables an accurate determination of both types of instability.

CIN tumors are not responsive to immune checkpoint therapies. It is possible that some MSI-positive tumors may not respond to immunotherapy due to the clonal divergence and presence of subclonal tumor cell populations with CIN characteristics. We are pursuing studies to determine if mixed MSI and CIN states alter immunotherapy response.

## Conclusions

Overall, we developed a new sequencing approach that determines MSI status based on all of the microsatellite classes, CIN status, and subclonal features. We found that the CIN phenotype was unexpectedly common in MSI tumors. Other studies validated this conclusion (Additional file 2: Table S21) [38–45]. Chromosome 8 shows alterations in the context of MSI and CIN. In addition, the microsatellite frameshift at exon 7 of *MSH3* and the degree of EMAST were associated with the mixed phenotype. This analysis of highly multiplexed microsatellites provided better quantitative accuracy and distinguished MSI tumors with distinct characteristics in mutation patterns in comparison to MSS tumors.

## Supplementary Information

**Additional file 1.** Supplementary Method, Supplementary Figures S1 – S16.

**Additional file 2.** Supplementary Tables S1 – S20.

**Additional file 3.** Sequences of OS-Seq primer probes.

**Additional file 4.** Log2 copy number ratio per gene from the targeted ultra-deep sequencing.

## Data Availability

The targeted and whole genome sequence data are available at the database of Genotypes and Phenotypes (dbGaP), accession number: phs001914.v1.p1 [52], https://www.ncbi.nlm.nih.gov/projects/gap/cgi-bin/study.cgi?study_id=phs001914.v1.p1.

## Abbreviations

CE: Capillary electrophoresis
CGH: Comparative genomic hybridization
CIN: Chromosome instability
CIN-B: Chromosome instability broad
CIN-F: Chromosome instability focal
CIN-L: Chromosome instability low
CN: Copy number
CRC: Colorectal carcinoma
CS: Chromosomally stable
EMAST: Elevated microsatellite alterations at selected tetranucleotide repeats
FISH: Fluorescent in situ hybridization
HMW: High molecular weight
IHC: Immunohistochemistry
Indel: Insertions or deletions
MMR: Mismatch repair
MS: Microsatellites
MSI: Microsatellite instability
MSI-H: Microsatellite instability high
MSI-L: Microsatellite instability low
MSS: Microsatellite stable
NGS: Next-generation sequencing
OS-Seq: Oligonucleotide-selective sequencing
R1: Read 1
R2: Read 2
STRs: Short tandem repeats
TCGA: The Cancer Genome Atlas Project
WGS: Whole genome sequencing
